# Olfactory testing in consecutive patients referred with suspected dementia

**DOI:** 10.1186/s12877-017-0516-2

**Published:** 2017-06-20

**Authors:** Ib Thrane Christensen, Elna-Marie Larsson, Ida E. Holm, Ole B.F. Nielsen, Stig Andersen

**Affiliations:** 1 Department of Geriatric Medicine, Aalborg University Hospital, Hobrovej 18-22, 9000 Aalborg, Denmark; 2 Department of Radiology, Aalborg University Hospital, Aalborg, Denmark; 3 Department of Pathology, Aalborg University Hospital, Aalborg, Denmark; 4 Department of Clinical Medicine, Aalborg University, Aalborg, Denmark

**Keywords:** Dementia, Alzheimer’s disease, Cognitive impairment, Olfactory dysfunction, Pocket smell test, Blinded study, Cuisine study

## Abstract

**Background:**

Alzheimer’s disease (AD) is the most common cause of dementia and early and accurate diagnosis is important. Olfactory dysfunction is an early sign of AD. The contribution by test of olfactory function has been surveyed in AD vs a line of conditions but remains to be settled in the workup of unselected patients referred with suspected dementia.

**Methods:**

We performed a two-step investigation: first, a comparative study of healthy controls and probable AD patients to test the applicability of the chosen scents (cuisine study); second, a study of consecutive patients referred to our geriatric outpatient clinic for suspected dementia with the investigating personnel blinded to the results of the Olfactory Test (blinded study).

**Results:**

The sum of scents detected discriminated patients with probable AD from controls in the cuisine study (*n* = 40; *p* < 0.001; area under ROC curve 0.94). In the blinded study (*n* = 50) the diagnosis was probable AD in 48%, minimal cognitive impairment in 24%, vascular dementia in 8%, alcohol induced impairment in 12%, depression in 4%, and Parkinson’s disease and Lewy body dementia in 2%. Area under the ROC-curve was 0.67. The odds ratio for probable AD with 2+ smell errors was 12 (95%-CI: 1.3–101; *p* = 0.026 (reference 0–1 smell errors)) age adjusted. None in the AD group had zero smell errors (Negative Predictive Value 100%).

**Conclusion:**

Olfactory testing may support to dismiss the diagnosis of probable AD in the workup of a mixed group of patients referred with cognitive impairment. Still, it had a low sensitivity for probable AD.

## Background

Alzheimer’s disease (AD) is the most common form of dementia [1]. It is characterized clinically by memory deficit followed by cognitive decline [1, 2]. Early diagnosis is important to prompt treatment and measures to aid patient and family caregivers [3] who may require support in their coping and understanding [4]. Hence, the quest for diagnostic precision for AD is advancing to include advanced genetic biomarkers in blood [5], markers in cerebrospinal fluid [6], and clinical tests such as recognition of emotions in facial expressions [7] to support early and accurate diagnosis of probable AD.

Olfactory information is processed in the medial temporal lobe and olfactory dysfunction relate to the extent of neuropathological changes in neurodegenerative diseases [8]. Olfactory dysfunction occurs in old age [9], but the age-related decline in olfactory function is accelerated in patients with AD and Parkinson’s disease (PD) [10, 11]. The ability to identify different odours is altered in the early stages of AD [12] and testing of olfactory function has been suggested as a diagnostic tool for AD from the early stages [12–15]. Furthermore, studies support the use of scent identification tests to aid the distinction between AD and depression [16] and vascular dementia [17].

Several commercially available tests of olfaction have been developed. They vary in number and quality of smells adjusted to cuisine and cultural differences [15, 18–20]. While the US population is familiar with root beer, pickles and gingerbread Europeans are not. Hence, different odour identification tests have been developed for use in the US [15, 19, 20] and in Europe [18, 20].

This led us to first validate the ability of six chosen scents to separate patients with probable AD from healthy controls in a simple and quick scratch-and-sniff test. Subsequently, we applied this technique in a convenience study to test the usefulness of the test to discriminate probable AD from non-AD among consecutive patients referred with cognitive decline when the health care professional involved in the diagnostic work-up was blinded to the results of the olfactory test.

## Methods

The study consisted of two parts. First, we conducted a cuisine pilot-study to validate the ability of selected scents to be detected by patients with AD and by healthy controls in Denmark. Second, we conducted a study of patients referred consecutively to our geriatric outpatient clinic for evaluation of cognitive decline with the clinicians blinded to the olfactory testing results.

### Cuisine study

Participants were recruited from the outpatient clinic at the Geriatric Department. The controls were volunteers from the local elderly community and healthy partners of the patients, and they were matched by gender.

Exclusion criteria included a history of nose-throat pathology with increasing sinusitis or chronic sinusitis, a flue condition, previous brain trauma, concussion of the brain with unconsciousness, and cerebral surgery.

### Blinded study

Participants were consecutive patients referred to the geriatric outpatient clinic at Aalborg University Hospital for evaluation of cognitive decline. Inclusion terminated at fifty patients. Exclusion criteria were similar to those applied in the cuisine study.

### Test of olfaction

We used a scent test with a scratch and sniff technique. It encompassed pads that release odours when scratched. The scent test was a Pocket Smell Test (PST) (Sensonic P.O.B. 112, Hadom High, New Jersey, US). Each PST included three different scents. Two different PSTs were used and each patient was exposed to six different scents. We chose PSTs with six different scents that had an international applicability (citrus, lilac, smoke, peanut, menthol, paint thinner).

Subjects were kept in a scent free room for at least 15 min prior to the test.

The test was appropriate for self-administration. However, short term memory is limited in these patients and a study-nurse scratched the pads and read the choices aloud in order to reduce cognitive load and to standardize administration as the focus was on providing the best environment for the patients to do the test. In the case of uncertainty the test was repeated and the patient was given one additional opportunity to complete the test.

Each smell was evaluated in two steps. *First step*: is there a smell yes/no. If no, then the participant did not pass. *Second step* consisted of four choices of which one matched the odour. The participant passed only if the correct smell was reported. Declining any of the four smells was recorded as not passing the test. Thus, we did not apply forced choice.

The result of the olfactory test (OT) was blinded to the examining doctor and nurse in the blinded study. A study nurse performed the test prior to the additional evaluation and this nurse was not involved in the subsequent work-up of the patient. The test results were kept unveiled until termination of the study.

### Clinical evaluation

All patients underwent clinical evaluation that included height, weight, routine laboratory tests, ECG, Folstein mini-mental state evaluation (MMSE test), geriatric depression scale (GDS), CT-scan of the brain, and history taking to evaluate the cognitive deficit. MMSE and GDS were performed at the first visit to the outpatient clinic. Final evaluation that led to the diagnosis was performed at a follow-up visit. As the study was set up to reflect the clinical practice patients were diagnosed with probable AD according to the ICD-10 criteria supported by the criteria set up by Goutie [21]. Diagnosis was made without knowledge of OT results.

All patients were examined with CT of the brain without contrast injection with a 5–10 mm slice thickness. An experienced neuro-radiologist (EML) evaluated all CT images and recorded cerebral infarctions and cortical atrophy prior to the clinical and biochemical evaluation.

Ethical approval was obtained from the Ethics Committee for Viborg and Nordjyllands Counties (VN-20060056) before commencement of the study and all participants gave written informed consent.

### Statistical analysis

Mean values were compared using Mann-Whitney *U* test and correlations were tested using Spearman’s rho. Proportions were compared using chi-squared test or Fischer exact test if groups were small. ROC curve was produced and the accuracy of the test was interpreted as excellent, good, fair, poor, and fail with an area under the curve (AUC) of >0.9, 0.8–0.9, 0.7–0.8, 0.6–0.7 and 0.5–0.6 respectively. AD was entered as dependent variable in logistic regression with age and OT results entered as explanatory variables. Data were processed and analysed using Corel Quattro Pro 8 and the statistical package for the social sciences version 13.0 (SPSS Inc., Chicago, Illinois). A *p*-value of less than 0.05 was considered significant.

## Results

The cuisine study consisted of 20 patients with probable AD and 20 healthy controls with similar gender distribution (Table 1). Patients were 5.5 years older than controls. The blinded study included 50 patients with suspected dementia. This group displayed equal gender distribution between those diagnosed with AD compared to those with cognitive impairment of other origin (non-AD) (Table 1). The patients with AD were older than non-AD patients. Two AD patients had no scent registration. All other AD patients confirmed some scent registration when exposed.

**Table 1:** Participant characteristics in a study comparing patients with probable Alzheimer’s disease (AD) and controls (cuisine study) and a study of patients referred for workup of suspected dementia blinded to the results of olfactory testing (blinded study)

	Cuisine Study			Blinded Study		
	AD	Controls	*p*-value	AD	not AD	*p*-value
Gender						
Men	7	7	ns*	12	13	ns*
Women	13	13		12	13	
Age (mean) (years)	80.9	75.4	0.001**	81.9	76.5	0.015**
(range)	(72–88)	(69–84)		(67–91)	(58–90)	

*Chi-squared test

**Mann-Whitney U test

### Cuisine study

**Fig. 1:**
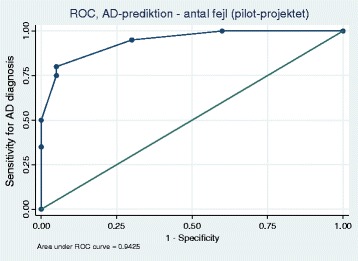
ROC curve from the cuisine study on the sensitivity and specificity of olfactory testing when comparing probable Alzheimer’s disease with healthy controls

AD patients scored a markedly higher number of errors on the OT compared to controls in the pilot study with an area under the ROC curve of 0.94 (Fig. 1). An average of 10.5 AD patients detected correct smells compared to 16.8 healthy controls (*p* = 0.005). All individual scents differed between AD and non-AD except lilac (Table 2).

**Table 2:** Number of errors for each scent among controls, Alzheimer’s disease (AD) and other causes of cognitive impairment

	Cuisine Study^a^			Blinded Study^b^		
	AD^c^	Controls	*p*-value^d^	AD^c^	non-AD^c^	*p*-value^d^
	*n* (%)	*n* (%)		*n* (%)	*n* (%)	
Citrus	14 (70)	4 (20)	<0.01	15 (62)	10 (38)	ns
Lilac	10 (50)	9 (45)	ns	15 (62)	15 (58)	ns
Smoke	12 (60)	0 (0)	<0.001	9 (37)	8 (31)	ns
Peanut	12 (60)	5 (25)	<0.05	13 (54)	10 (38)	ns
Menthol	11 (55)	1 (5)	<0.001	16 (67)	13 (50)	ns
Paint thinner	12 (60)	0 (0)	<0.001	18 (75)	8 (31)	<0.01

^a^Comparing patients with probable Alzheimer’s disease and helathy controls.

^b^study of consecutive patients referred for suspected dementiat with the results of test of olfactory function blinded to the diagnosis

^c^Alzheimer’s disease

^d^Chi-squared test or Fishers exact test when *n* < 5

ns *p*-value >0.05

### Blinded study

**Fig. 2:**
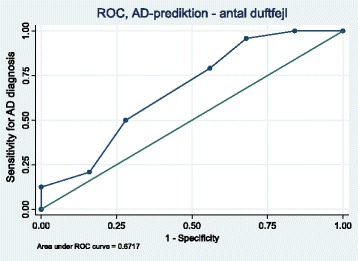
ROC curve to depict the sensitivity and specificity of olfactory testing to detect probable Alzheimer’s disease in the blinded study of consecutive patients referred to a geriatric outpatient clinic for workup of suspected dementia

The detection of one single scent differed clearly between AD and non-AD patients while all individual scents displayed a slightly higher number of errors in AD patients compared to non-AD patients (Table 2). The sum of scent errors differed between AD patients and those with other causes of cognitive impairment (*p* = 0.024) (Table 3). The contribution by OT to the classification as probable AD and non-AD is illustrated in Fig. 2 (area under the ROC curve 0.67). The test showed the higher accuracy at the extremes and the lower accuracy at the central area.

**Table 3:** Findings among 50 consecutive patients referred for suspected dementia to the outpatient clinics at Department of Geriatric Medicin, Aalborg University Hospital

	*n*	Age	MMSE score^a^	GDS^b^	Cerebral	Cortical	Olfactory test^e^
		years			infarctions^c^	atrophy^d^	Errors
		mean (range)	mean (range)	mean (range)	*n*	*n*	mean (range)
Alzheimers disease	24	81.9 (67–91)	22.5 (11–29)	2.1 (0–7)	6	9	3.6 (1–6)
Not Alzheimers disease	26	76.5 (58–90)	24.0 (10–30)	3.5 (0–14)	9	4	2.5 (0–5)
Minimal cognitive impairment	12	81.3 (72–90)	24.4 (10–29)	2.7 (0–9)	5	2	2.0 (0–5)
Lewi body dementia	1	77.0	24.0	4.0	0	0	3.0
Vascular dementia	4	79.2 (73–84)	22.5 (13–27)	1.5 (1–2)	3	0	3.0 (1–4)
Parkinson’s disease	1	63.0	28.0		1	0	3.0
Depression	2	79.5 (79–80)	27.5 (25–30)	2.0 (0–4)	0	1	1.5 (0–3)
Alcohol induced impairment	6	66.2 (58–78)	22.5 (12–30)	4.8 (1–14)	0	1	3.2 (0–5)

^a^Folstein mini-mental state evaluation (MMSE test)

^b^Geriatric Depression Scale. Data missing for a patient with Parkinson’s disease

^c^ > 3 mm on CT of the brain

^d^Sulci widening grade 3

^e^Number of incorrect answers of 6 possible in the Pocket Smell Test

None of the AD patients detected all scents correctly (Table 4, *p* = 0.045) and none without scent errors had AD (Table 4, *p* = 0.10). The odds ratio for AD in patients with 2 or more smell errors was 12 (95%-CI: 1.3–101; *p* = 0.026) after adjusting for age (Table 5). Positive predictive value for AD was 100% with maximum number of smell errors in this population while negative predictive value for AD was 100% with no smell errors in this population (Table 5).

**Table 4:** Number of smell errors as detected in patients with probable Alzheimers Diasease (AD) or non-AD patients in the blinded study

	AD	non-AD
	n^a^	n^a^
Number of smell errors:		
0	0	4
1	1	5
2	4	3
3	7	7
4	7	3
5	2	4
6	3	0

^a^Number of patients

**Table 5:** Prediction of the diagnosis of Alzheimer’s disease in an out-patient population referred for evaluation of dementia (blinded study)

	Alzheimers disease							
	*P* ^a^	OR^b^	95-CI	*P* ^c^	OR^c^	95-CI	PPV (%)^f^	NPV (%)^g^
Number of smell errors								
1+ (0 reference)	0.045	na^d^			na^d^		52	100
2+ (0–1 reference)	0.011	12.2	1.4–105	0.026	11.6	1.3–101	58	90
3+ (0–2 reference)	0.059	3.3	0.9–11.4	0.078	3.3	0.9–12.1	58	71
4+ (0–3 reference)	0.093	2.7	0.8–8.8	0.055	3.5	1.0–12.6	63	61
5+ (0–4 reference)	0.62	1.4	0.3–6.2	0.73	1.3	0.3–6.6	56	54
6 (0–5 reference)	0.10	na^e^			na^e^		100	55

^a^Chi squared test, Fisher’s exact test if *n* < 5

^b^Univariate logistic regression

^c^Multivariate model adjusted for age (<80y) and MMSE (<20)

^d^Not applicable: 4, all non-AD

^e^Not applicable: 3 reference, all AD

^f^Positive predictive value, %

^g^Negative predictive value, %

OT associated with MMSE score (Spearmann’s rho −0.42, *p* = 0.002) and MMSE score associated with OT in non-AD patients (*p* = 0.029) while not in AD patients (ns). The number of scent errors differed with gender in non-AD patients (median in men/women 1.0/3.0; *p* = 0.022) while not in AD-patients (men/women 4.0/3.0; *p* = 0.71). Geriatric Depression Score may be lower in AD patients than in non-AD patients (*p* = 0.11; Table 3). AD and non-AD patients did not differ markedly in the number of subjects with cerebral infarctions (*p* = 0.47) or cerebral atrophy (*p* = 0.15) (Table 3).

### Cuisine and blinded study

All patients with probable AD had high numbers of errors on OT and this did not differ between patients in the blinded study and the cuisine study (59.2 vs. 59.7%, ns). Patients with cognitive impairment of other cause than AD had more errors on the OT than did the healthy controls (41.0 vs 15.8%, *p* < 0.001).

## Discussion

We made a two-step investigation. First, we validated the applicability of a commercially available 6-item PST with an international applicability in an elderly population in Denmark. We found that five of the six scents individually contributed markedly to the OT in making a distinction between probable AD patients and healthy controls, and the area under the ROC curve suggested excellent distinction between the two groups. Second, we tested the usefulness of this OT in a blinded study among consecutive patients referred with suspected dementia. The accuracy of the OT was poor in the blinded study as evaluated from the area under the ROC curve. Still, we found that the OT provided some contribution to the diagnostic workup of patients with suspected dementia in supporting to dismiss the diagnosis of probable AD in the group with a single or no errors on the OT. Interestingly, this was further supported by the fact that all 46 patients with probable AD in both studies had one or more errors in the six scents included in this PST, and none of the non-AD patients or controls had all scents incorrect.

Cognitive impairment is common in old age and AD is the most frequent form of dementia. Early diagnosis of probable AD is crucial to support intervention for the benefit of patient and family [3, 4]. However, the pathology of AD may be present decades before a clinical diagnosis of dementia [2, 22]. Early changes in AD include smell deficits [12] but smell deficits are seen in a number of other neurodegenerative disorders [10]. Yet, a recent functional MRI study in AD patients confirmed a degeneration of neural structures responsible for olfactory function [23]. This supports that a central component is predominant in the damage to the smell pathways in AD patients and that loss of smell is an early sign of AD [12] that may be present before the diagnosis of probable AD. It has been suggested that OT should be used in clinical routine for early identification of progression of the decline from mild cognitive deficits to probable AD [12–15, 22]. We took a different view and studied its applicability in a routine clinical setting of patients referred consecutively for suspected dementia.

Cognitive impairment may have several causes such as depression, infection, and the use of multiple drugs. In fact, one of our participants had a MMSE score of 10 at the first visit but after tapering of morphine the cognitive impairment decreased markedly and the patient ended up with the diagnosis of MCI. Thus, the mean MMSE score went up to 25.7 points for the MCI group when testing only the remaining patients, of which the scores ranged from 21 to 29.

Our data confirm previous findings that patients with probable AD have a markedly reduced ability to recognize scents [11–15, 18]. A study from Norway found similar results to our cuisine study when testing AD patients and healthy controls using the Brief Smell Identification Test (B-SIT) with twelve different scents [24]. The highly statistically significant results of our cuisine study suggest that a lower number of scents will suffice.

Test of olfactory function may face practical problems such as stability of the scent. We chose a scratch and sniff technique from the company Sensonic [19]. Tests were available with different elements, and we chose two PSTs that each included three scent pads. It has been demonstrated that a single PST can aid a distinction between AD and major depression [16, 17] and we used two different PSTs to accommodate the number of tests aimed for.

Patients in the blinded convenience study all had some degree of cognitive impairment. We found limited specificity of the OT in the workup of the group of patients with mixed cause of cognitive impairment. Still, the difference in the association between MMSE score and OT in AD versus non-AD patients with cognitive impairment is consistent with a difference in olfactory function between these patients. This was also seen with the gender difference in scent detection between AD and non-AD patients. OT was not sufficiently accurate to be used as a sole diagnostic tool for diagnosis of probable AD, but six errors could be seen as supporting probable AD with a positive predictive value for AD of 100% in our data. Interestingly, taking the opposite view, the six-item OT may suggest dismissing the diagnosis of probable AD among those who had no errors in our study as we found a negative predictive value of 100% in this group. This is in keeping with three studies testing smell among AD patients using the PST [25] and the B-SIT [24, 26]. Hence, having no smell-error supports dismissing the diagnosis of probable AD.

Odour identification depends on cultural and cuisine areas [18, 24] and the relevance of odours may need validation prior to implementing an OT. However, similarities exist within cultural and geographical areas and the distinct value in five of six individual scents seen in our study suggests that these may be applicable to other countries in northern Europe.

Performance of the scent identification tests poses risk of error. Adaptation and cross-adaptation may compromise the validity of the test and the intensity of the scent exposure should not be too strong. Also, the exposure should be short and it is important for the scent quantity and quality to be standardised. These requirements were accommodated by the scratch-and-sniff technique. Furthermore, there is a risk of adaptation with an increase in the number of smells tested and we chose to use only six smells. This was supported by an impression of a decreasing focus on the smell test with increasing number of tests. The use of a brief test was further justified by the fact that patients with dementia have a limited window of short-term memory. This further encouraged the procedure of having the study nurse scratch and read aloud the four possible choices. Finally, we kept the subjects in a scent neutral area for at least 15 min before scent exposure to minimise interference with other scents and adaptation to support the validity of our findings.

Forced choice of odour identification has been used [19]. This causes one in four answers to be correct due to chance. Patients who declined to guess odour comprised a group that performed similarly to the functionally anosmic group in the study by Damholdt [27]. Thus, we chose not to use forced choice but rather categorized the test results in these patients as ‘fail’. This occurred in two AD patients. Also, some patients were in doubt and the scratch, sniff and read aloud procedure was repeated. A continued recognition of a scent was common in these patients even though they were not able to name it or identify it among the four possibilities given.

The ability to recognize scents is stabile from around the age of 20 years to between 55 and 60 years of age. Thereafter, the ability to recognize scent is gradually reduced [8, 9, 25]. Hence, we adjusted for age in our analysis.

## Conclusions

Patients with probable AD with early olfactory impairment make up a considerable proportion of patients referred for evaluation of suspected dementia. Hence, a brief, simple, convenient, and cheap test of olfactory impairment is warranted. These characteristics apply to the PST that has been shown here to add information in the workup of a group of patients referred with cognitive impairment. Its simplicity supports its use in routine clinical practice. The results suggest that test of olfactory function has the potential to dismiss the diagnosis of probable AD. Finally, the difference in accuracy between the cuisine study and the blinded study emphasise the importance of putting a clinical test to the test in the clinical setting intended for its use.

## Abbreviations

AD: Alzheimer’s Disease
B-SIT: Brief Smell Identification Test
CI: Confidence Interval
CT: Computer Tomography
ECG: Electrocardiograph
GDS: Geriatric Depression Scale
MCI: Mild Cognitive Impairment
MMSE: Mini-Mental State Evaluation
NPV: Negative Predictive Value
OT: Olfactory test
PPV: Positive Predictive Value
PST: Pocket Smell Test
ROC: Receiver Operating Characteristic
